# Comparison of alternating home telemedicine consultations with regular face to face consultations in type 1 diabetes

**DOI:** 10.1186/1687-9856-2015-S1-P27

**Published:** 2015-04-28

**Authors:** Peter W Goss, Jennifer L Goss, William E Goss

**Affiliations:** 1 Gippsland Paediatrics Diabetes Unit, VIC, Australia; 2 School of Medicine, Monash University, VIC, Australia

## Aims

To compare a model of alternate home videoconference consultations/face to face consultations with regular face to face consultations in young rural patients with Type 1 Diabetes Mellitus (T1DM).

## Methods

A 12 month non randomized controlled trial was performed in 2013 comprising a cohort of children, adolescents and young adults with T1DM from the immediate local region compared with a similar cohort from a region greater than 70 Km away. The local cohort continued with 3 monthly appointments and extra visits as required between appointments. The distant cohort had 6 monthly face to face consultations, alternating with 6 monthly formal videoconference consultations to their homes. Extra visits were also managed via videoconference.

Outcome was measured by comparison of HbA1c between the two groups before during and after the intervention. Missed or rescheduled visits were compared. A patient satisfaction survey was performed and logistic issues were described from both the patient and medical team perspective.

## Results

30 patients (mean age 18.3 years) in the control group (mean HbA1c 8.4%) were matched with 29 patients (mean age 17.2 years) in the intervention group (mean HbA1c 8.3%) (NS). During the intervention period, the glycaemic control in both groups deteriorated slightly (control 8.7%, intervention 8.5%) (p=0.31) Upon return to regular 3 monthly appointments, HbA1c was 8.4% (control) and 8.6% (intervention). Missed or rescheduled appointments occurred more in the telemedicine group.

Patient satisfaction was strong for 4 measures of convenience (time of day, home location, accessibility to other parent/partner and time off school/work) but with major inconvenience accessing HbA1c testing. The major disincentives were lack of personal interaction and more difficulty discussing difficult issues. The major issues for the medical team were reduced ability to read patient’s and parent’s emotions because of technology and less commitment to appointments by some families.

## Conclusion

Telemedicine consultations to home are well accepted and convenient for rural families and young adults with T1DM but are associated with more difficulty accessing HbA1c tests, more missed appointments and more difficulty reading emotional cues during consultations. Glycaemic control did not improve. Catch up videoconferences between appointments were very well accepted. Replacing face to face consultations with direct home videoconference should be done with caution.

